# High-Frequency Ultrasonography and Evaporimetry in Non-invasive Evaluation of the Nail Unit

**DOI:** 10.3389/fmed.2021.686470

**Published:** 2021-06-14

**Authors:** Marta Szymoniak-Lipska, Adriana Polańska, Dorota Jenerowicz, Adam Lipski, Ryszard Żaba, Zygmunt Adamski, Aleksandra Dańczak-Pazdrowska

**Affiliations:** ^1^Department of Dermatology, Poznan University of Medical Sciences, Poznan, Poland; ^2^Department of Dermatology and Venereology, Poznan University of Medical Sciences, Poznan, Poland; ^3^Department of Urology, Poznań University of Medical Sciences, Poznan, Poland

**Keywords:** HF-USG, High frequency ultrasonography, TOWL, transonychial water loss, nail plate thickness, nail, nail unit

## Abstract

**Background:** The nail unit (NU) is a complex structure that performs a number of functions, including protection, defense, manipulation, and palpation. Non-invasive research methods can facilitate the recognition of NU structure and function. Evaporimetry and HF-USG due to their availability of equipment and low research costs seem to be particularly noteworthy, but so far have been assessed to a limited extent. The aim of the presented study was to check the usefulness of TOWL and HF-USG in examination of NU.

**Materials and Methods:** A total of 58 volunteers aged 25–65 years (mean age: 41 ± 10.16 years) were qualified for the study. The subjects did not present symptoms of clinically evident onychopathy and did not suffer from any dermatoses associated with lesions occurring within the NU. Additionally, the patients did not suffer from systemic diseases that could affect NU (including heart, lung, and endocrine diseases). In all volunteers, the measurement of TOWL and 20 MHz ultrasonography [high-frequency ultrasonography (HF-USG)] with the special emphasis on determination of nail plate thickness were performed.

**Results:** Analysis of 464 HF-USG images revealed that the nail plate presented as two hyperechoic, parallel streaks (railway sign) with a linear hypoechoic middle layer between them. Matrix was visualized as a hypoechoic structure with blurred boundaries, mostly within the fourth and fifth fingers and more often in women. We found statistically significant correlations between the type of a finger and the thickness of the nail plate both in the entire study group and taking into account gender. In the dominant hand, the results were *r* = −0.341; *p* < 0.001; *r* = −0.417, *p* < 0.001; and *r* = 0.337; *p* = 0.001 (for the whole group, for women, and for men, respectively). In the non-dominant hand, the results were *r* = −0.465; *p* < 0.001; *r* = −0.493, *p* < 0.01; and *r* = −0.503; *p* < 0.01 (for the whole group, for women, and for men, respectively). There were statistically significant differences in the thickness of the nail plates of the corresponding types of fingers between female and male NUs. Statistically significant correlations were found between the type of a finger and the TOWL value in the whole group and taking into account gender (*p* < 0.05), except for the non-dominant hand in men. There were no statistically significant differences in the TOWL values of the corresponding types of fingers between male and female NUs (*p* > 0.05). There was no statistically significant correlation between the TOWL value and the nail plate thickness in any of the tested NUs, apart from the one statistically significant correlation in nd5 (*r* = 0.390, *p* = 0.021).

**Conclusions:** To sum up, non-invasive methods, such as HF-USG and TOWL, enable assessment of the NU and are useful in examination of its structure and function. HF-USG shows characteristic elements of NUs that can be distinguished because of differences in their echogenicity. The thickness of the nail plate and TOWL depend on the type of finger, and show a relationship with gender.

## Introduction

The nail unit (NU) is a complex structure that performs a number of functions, including protection, defense, manipulation, and palpation (1). Nowadays special attention is also paid to the aesthetic function of NU (2), and deterioration in its appearance may negatively affect the quality of life (3). For those reasons, diseases of NU seem to be hot topics for researchers around the world (4, 5). There is a significant tendency to look for non-invasive research methods that facilitate the recognition of NU structure and function. Among these methods, evaporimetry and HF-USG seem to be particularly noteworthy, due to the availability of equipment, and low research costs that so far have been assessed to a limited extent (6).

Transepidermal water loss (TEWL) measurement is used to indirectly assess the function of the epidermal barrier function and is applied mostly in studies conducted among patients with atopic dermatitis (AD) (7–9). The modification of this procedure adapted to NU is a transonychial water loss (TOWL). TOWL values have been reported in single studies and are many times higher than the TEWL values, which can be related to NU composition (10). The possibility of imaging NU in real time can be achieved using ultrasound examination (USG). With the application of frequencies dedicated for dermatological applications [high-frequency ultrasound (HF-USG)] (11), there is the possibility to visualize the NU structure. To date, in single reports, NU pathological processes like abscesses, hematomas (12), glomeruli, and mucous cysts were described with the use of HF-USG, and the utility of this method in differentiation of psoriasis (13) and onychomycosis (14) was presented. However, studies evaluating usefulness of HF-USG in examination of complete finger NU structure in regards to sex, age, and dominant hand are still lacking. The relationship between TOWL value and thickness of the nail plate was assessed only to a small extent in few reports (6, 15, 16), using a variety of research methodology. Thus, there is a need for detailed, global ultrasonographic assessment of NU, the knowledge of which could translate into a better understanding of the pathological processes occurring within this appendage.

The aim of the presented study was to explore the utility of TOWL and HF-USG in examination of NU as well as to obtain the ultrasonographic features of NU with special emphasis on non-invasive measurement of the nail plate thickness.

## Materials and Methods

### Examined Group

A total of 58 volunteers aged 25–65 years (mean age: 41 ± 10.16 years) were qualified for the study. Thirty-five women (mean age: 41.49 ± 9.80 years) and 23 men were examined (mean age: 40.26 ± 10.86 years). All of the participants have given their written consent. The subjects did not present symptoms of clinically evident onychopathy and did not suffer from any dermatoses associated with lesions occurring within the NU. All subjects were right-handed and non-smokers. In two cases, two NU of the thumbs were excluded from the study due to the condition after mechanical trauma.

The tests were performed in standardized conditions at the temperature of 20–24°C. At least 30 days prior to examination, volunteers had to stop using any nail polish or varnish and performing manicure, except nail clipping. Subjects were asked not to use detergents and disinfectants for a minimum of 20 min before tests. First, TOWL measurement was performed, followed by HF-HSG. The above order was determined by necessity to use a medium in the form of an ultrasound gel for HF-USG examination. We were aware that its application to the nail plate could cause a change in NU hydration and make TOWL results unreliable.

The letter designation was adopted for the description of NU: “d” for the finger of the dominant hand and “nd” for the non-dominant one. The number describes the type of finger, and so the number (1), corresponded to the thumb; (2), the index finger; (3), the middle finger; (4), the ring finger; and (5), little finger.

### HF-USG

The HF-USG examination was performed with the use of The Dermascan C device (version 3) by Cortex Technology (Hadsund, Denmark) equipped with a linear head 20 MHz (11). The probe was covered with a layer of ultrasound gel and then placed on the NU to obtain sagittal sections. The transducer was positioned in the midline to include the proximal nail fold and 1/2 proximal to the nail plate in the imaging. The axial resolution was 80 μm, and the lateral resolution was 200 μm. The ultrasonic wave propagated through the tissues at a speed of 2,480 m/s. The B-mode presentation was used to qualitatively evaluate the images. The A-mode presentation was used to carry out the measurements of the thickness of the nail plate, which was measured in each examined finger three times in the area between the proximal 1/3 and middle 1/3. Then, the arithmetic mean was calculated.

### TOWL

TOWL measurements were conducted using a Tewameter^®^ TM 300 Courage-Khazaka (Köln, Germany), with a reduction diaphragm recommended by the manufacturer (diameter 2 mm) for testing small areas (Z00812) according to the guidelines originally established for TEWL (17). The Tewameter^®^ TM 300 was connected to the Cutometer^®^ MPA 580. The device was placed in the middle on the nail plate within its proximal 1/3 and central part. An attempt was made to achieve the comparable pressure. In case of each examined NU, 20 measurements were performed at 1 s intervals. An arithmetic mean was then calculated. The results were presented in conventional units (c.u.).

### Statistical Analysis

For the values of parameters such as plate thickness and TOWL, appropriate descriptive statistics were determined: arithmetic mean with the corresponding standard deviation, minimum, and maximum values. The differences between the groups were assessed using the non-parametric Mann–Whitney *U* test. The relationships between the parameters were investigated using Spearman's correlation. Values at *p* < 0.05 were considered statistically significant. The calculations were made with the use of the STATISTICA v 13.3 statistical package.

## Results

### Analysis of HF-USG Results

Finally, HF-USG 464 images of NU were taken into consideration, which was related to the repeated unsuccessful attempts to visualize NU in the thumbs. The difficulties were associated with the inability to fit an ultrasound head for anatomical curvatures of NU within d1 and nd1 and the gained images had insufficient quality. Therefore, analysis of them was abandoned.

**Figure 1:**
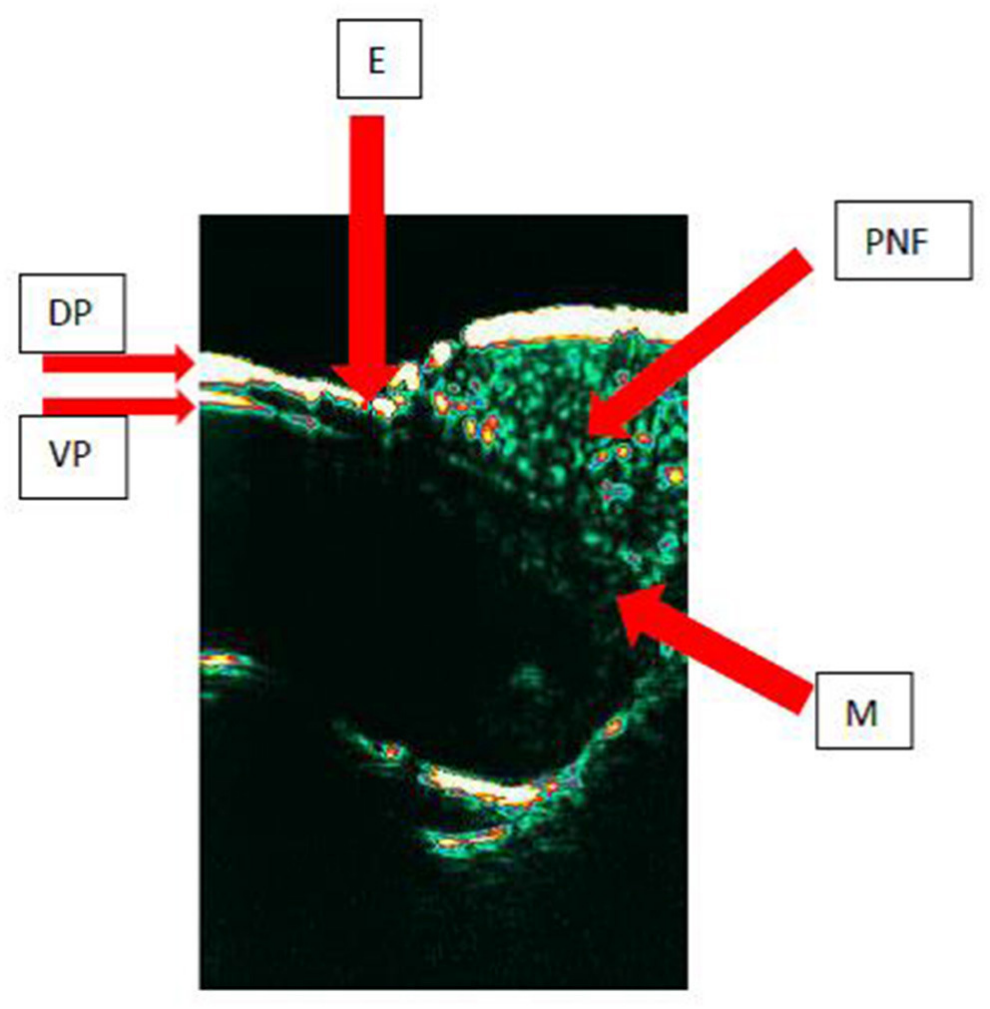
The HF-USG image of the nail unit.

In all analyzed fingers, the nail plate presented as two hyperechoic, parallel streaks (railway sign). Between them, a linear hypoechoic middle layer was visible. The abovementioned hyperechoic bands were, respectively, the dorsal and ventral lamina of the nail plate. The cuticle was located proximally to the described structures and its echogenicity was similar to the echogenicity of the dorsal and ventral plates. Moving further, proximal nail fold, with an echogenicity lower than the mentioned dorsal and ventral plates, occurred. Matrix was visualized as a hypoechoic structure with blurred boundaries, adjacent to the nail fold and the proximal part of the abdominal plate. It was visible in a minority of respondents, and the number of visualized matrixes varied in different types of finger. The matrix was seen more often in the fourth and fifth fingers and more often in women. The detailed percentage results of imaged matrixes are presented in Table 1. Figure 1 shows the sagittal section of HF-USG of the NU with its characteristic elements.

**Table 1:** Percentage results of imaged matrixes.

**Whole group**								
Type of finger	d2	d3	d4	d5	nd2	nd3	nd4	nd5
% of visible matrixes (%)	12	9	19	22	21	22	28	24
**Women**								
Type of finger	d2	d3	d4	d5	nd2	nd3	nd4	nd5
% of visible matrixes (%)	14	11	29	29	29	31	37	20
**Men**								
Type of finger	d2	d3	d4	d5	nd2	nd3	nd4	nd5
% of visible matrixes (%)	9	4	4	13	9	9	13	30

*nd, the finger of the non-dominant hand; d, the finger of the dominant hand; number, type of finger; i.e., 2, index finger*.

The average thickness of the nail plate in the whole group ranged from 0.36 mm in nd5 to 0.43 mm in nd2. The thinnest nail plate was observed in the group of women in nd5 (0.25 mm), and the thickest was observed in the group of men in nd2 (0.54 mm). The mean thickness of the nail plate in the group of women ranged from 0.35 mm in nd5 to 0.40 mm d2. The greatest thickness in this group was observed in nd2 (0.51 mm). The average thickness of the nail plate in the group of men ranged from 0.39 mm in nd5 to 0.45 mm in nd2. The lowest thickness in this group was noted in nd4 (0.30 mm). The thickness of nail plates is presented in Table 2.

**Table 2:** The thickness of nail plates in the whole group and in the group of women and men.

**Whole group**				
**Type of finger**		**Mean** **±** **SD [mm]**	**Min [mm]**	**Max [mm]**
2	D	0.42 ± 0.05	0.33	0.52
	Nd	0.43 ± 0.04	0.35	0.54
3	D	0.41 ± 0.05	0.30	0.51
	Nd	0.40 ± 0.05	0.29	0.50
4	D	0.39 ± 0.04	0.29	0.49
	Nd	0.38 ± 0.05	0.28	0.52
5	D	0.38 ± 0.05	0.27	0.48
	Nd	0.36 ± 0.04	0.25	0.45
**WOMEN**				
**Type of finger**		**Mean** **±** **SD [mm]**	**Min [mm]**	**Max [mm]**
2	D	0.40 ± 0.04	0.33	0.49
	Nd	0.41 ± 0.04	0.35	0.51
3	D	0.39 ± 0.04	0.30	0.46
	Nd	0.39 ± 0.04	0.29	0.48
4	D	0.37 ± 0.04	0.29	0.44
	Nd	0.37 ± 0.04	0.28	0.43
5	D	0.36 ± 0.04	0.27	0.44
	Nd	0.35 ± 0.04	0.25	0.42
**MEN**				
**Type of finger**		**Mean** **±** **SD [mm]**	**Min [mm]**	**Max [mm]**
2	D	0.45 ± 0.05	0.35	0.52
	Nd	0.45 ± 0.04	0.36	0.54
3	D	0.43 ± 0.05	0.34	0.51
	Nd	0.43 ± 0.04	0.36	0.50
4	D	0.41 ± 0.04	0.34	0.49
	Nd	0.41 ± 0.06	0.30	0.52
5	D	0.41 ± 0.04	0.33	0.48
	Nd	0.39 ± 0.03	0.33	0.45

*nd, the finger of the non-dominant hand; d, the finger of the dominant hand; number, type of finger; i.e., 2, index finger*.

We found statistically significant correlations between the type of a finger and the thickness of the nail plate both in the entire study group and taking into account gender. In the dominant hand, the results were as follows: *r* = −0.3; *p* < 0.001; *r* = −0.4, *p* < 0.001; and *r* = 0.3; *p* = 0.001 (for the whole group, for women, and for men, respectively). In the non-dominant hand, the results were *r* = −0.5; *p* < 0.001; *r* = −0.5, *p* < 0.01; and *r* = −0.5; *p* < 0.01 (for the whole group, for women, and for men, respectively). There were statistically significant differences in the thickness of the nail plates of the corresponding types of fingers between female and male NUs (Table 3).

**Table 3:** *p*-values for differences in the thickness of the nail plates of the corresponding types of fingers between female and male NUs.

**Type of finger**	**d2**	**d3**	**d4**	**d5**	**nd2**	**nd3**	**nd4**	**nd5**
*P*	<0.001	0.002	0.002	<0.001	<0.001	0.001	0.026	<0.001

*nd, the finger of the non-dominant hand; d, the finger of the dominant hand; number, type of finger; i.e., 2, index finger*.

There were no statistically significant differences in the thickness of the nail plates of the corresponding fingers of the dominant and non-dominant hand, both in the whole group and taking into account gender (*p* > 0.05).

The statistically significant correlation between the age and the thickness of the nail plate was observed only in the d5 fingers in the entire study group and in the group of men (*r* = −0.3, *p* = 0.027; *r* = −0.4, *p* = 0.043, respectively).

### Analysis of TOWL Values

The mean TOWL value in the entire group ranged from 5.02 c.u. in d1 to 7.41 c.u. in d5. The lowest measured TOWL value was recorded in the group of men in d1 (1.10 c.u.), and the highest value, also observed in the group of men, in nd3 (17.60 c.u.). The mean value of TOWL in the group of women ranged from 5.1 c.u. in d1 to 7.30 c.u. in d5. In this group, the minimal value of TOWL was observed in d1 (2.70 c.u.), and the highest value (12.80 c.u.) was obtained in d5. The mean value of TOWL in the group of men ranged from 4.90 c.u. in d1 to 7.58 c.u. in d5.

The TOWL value for each type of NU are presented in the Table 4

**Table 4:** The values of TOWL measurements in the whole group, in the group of women, and in the group of men.

**Whole group**				
**Type of finger**		**Mean** **±** **SD [c.u.]**	**Min [c.u.]**	**Max [c.u.]**
1	D	5.02 ± 1.50	1.10	8.00
	Nd	5.33 ± 1.72	2.90	11.60
2	D	6.13 ± 1.80	3.30	10.90
	Nd	6.41 ± 2.27	2.10	12.80
3	D	6.64 ± 2.70	2.30	16.80
	Nd	6.43 ± 2.39	3.10	17.60
4	D	6.74 ± 2.00	2.90	12.50
	Nd	6.52 ± 2.24	3.30	16.30
5	D	7.41 ± 2.50	3.10	13.30
	Nd	6.53 ± 2.13	1.80	11.80
**WOMEN**				
**Type of finger**		**Mean** **±** **SD [c.u.]**	**Min [c.u.]**	**Max [c.u.]**
1	D	5.10 ± 1.20	2.70	8.00
	Nd	5.39 ± 1.55	2.90	9.90
2	D	5.92 ± 1.73	3.70	10.60
	Nd	6.40 ± 1.93	3.30	11.50
3	D	6.36 ± 1.64	3.90	12.00
	Nd	6.07 ± 1.40	3.50	8.50
4	D	7.03 ± 1.95	3.60	12.50
	Nd	6.50 ± 2.02	3.30	10.70
5	D	7.30 ± 2.41	3.50	12.80
	Nd	6.61 ± 1.87	3.90	11.10
**MEN**				
**Type of finger**		**Mean** **±** **SD [c.u.]**	**Min [c.u.]**	**Max [c.u.]**
1	D	4.90 ± 1.91	1.10	8.00
	Nd	5.24 ± 1.99	3.00	11.60
2	D	6.46 ± 1.90	3.30	10.90
	Nd	6.43 ± 2.75	2.10	12.80
3	D	7.08 ± 3.80	2.30	16.80
	Nd	6.97 ± 3.36	3.10	17.60
4	D	6.31 ± 2.03	2.90	10.40
	Nd	3.55 ± 2.58	3.40	16.30
5	D	7.58 ± 2.67	3.10	13.30
	Nd	6.41 ± 2.51	1.80	11.80

*Nd, the finger of the non-dominant hand; d, the finger of the dominant hand; number, type of finger; i.e., 2, index finger; c.u., conventional units*.

Statistically significant correlations were found between the type of a finger and the TOWL value in the whole group and taking into account gender (*p* < 0.05), except for the non-dominant hand in men (*r* = 0.8, *p* = 0.068) (Table 5).

**Table 5:** Values of *r* and *p* for correlations between the type of a finger and the TOWL value.

**Dominant hand**	***R***	***P***
Whole group	0.3	<0.001
Women	0.4	<0.001
Men	0.3	0.003
Non-dominant hand	*R*	*P*
Whole group	0.2	0.002
Women	0.2	0.012
Men	0.8	0.068

There were no statistically significant differences in the TOWL values of the corresponding fingers of the dominant and non-dominant hand, both in the whole group and taking into account gender (*p* > 0.05). There were no statistically significant differences in the TOWL values of the corresponding types of fingers between male and female NUs (*p* > 0.05). The statistically significant correlation between the age and the TOWL value was observed only in the group of men in d3 finger (*r* = −0.5, *p* = 0.031).

### Analysis of Correlations Between HF-USG Results and TOWL Values

There was no statistically significant correlation between the TOWL value and the nail plate thickness in any of the tested NUs, neither in the whole group and in the group of men. The significant correlations were not detected in the majority of fingers in women's group, the only statistically significant correlation was pointed in nd5 (*r* = 0.4, *p* = 0.021).

## Discussion

To our knowledge, our study presents for the first time a detailed characteristic of a NU achieved with the use of non-invasive tools in subjects with no evident onychopathy, taking into account all fingers of both hands in relation to volunteers' gender and age.

### HF-USG

Similarly to previous studies (12), we found that the normal nail plate is a trilaminar structure with the first and third layer being hyperechoic, while the second layer is anechoic. The nail matrix is a hypoechoic area that is placed proximally within NU.

In our study, matrix was visible in a minority of respondents, and the number of visualized matrixes varied in different types of fingers. The matrix was seen more often in the fourth and fifth fingers and more often in women. It may be due to the fact that in these fingers, the nail plate is the thinnest and the visualization of deeper NU structures is easier. The other reason could be that the curvature of mentioned NUs is the slightest and USG probe adheres better, therefore giving higher chances to obtain good quality images. Unfortunately, in our study images gained from d1 and nd1 had insufficient quality, although repeated attempts to visualize these NU were performed. The difficulties were related to the limitations of our probe (inability to fit the ultrasound head for anatomical curvatures of thumbs). Finally, HF-USG 464 images of NU of fingers two to five were taken into consideration.

Literature data regarding NU USG in healthy volunteers are scarce and most researchers focus on NU pathologies (particularly psoriasis) (18–20). The pioneers of a nail plate thickness analysis using ultrasound transmission time were Finlay et al. (21). They compared acquired data with micrometer measurements and concluded that ultrasounds can be useful to determine nail thickness. In our study, we used A-mode to estimate the nail plate thickness. The average thickness of the nail plate in the whole group ranged from 0.36 mm in nd5 to 0.43 mm in nd2. Estimated borderline values suggest that the thinnest nail plate was observed in female volunteers in nd5 (0.25 mm), and the thickest was observed in males in nd2 (0.54 mm). Similar results were obtained by Wollina et al. (22)—according to their analysis, the thickness of the nail plate ranged between 0.481 mm (right hand thumb) and 0.397 mm (fifth finger of the left hand).

We observed statistically significant correlations between finger type and the thickness of the nail plate (in both the dominant and non-dominant hand) in the whole group and taking gender into account (*p* < 0.05). The average thickness of the nail plate was lower the further the finger was from the thumb. We assume that those observations arise from the more frequent use of the radially located fingers in everyday activities. The greater exposure to mechanical stimuli requires more effective protection of the fingertips and hence the appearance of thicker nail plates in index fingers than in the little fingers.

In our study, gender-related differences between nail thickness were observed—men presented with thicker nail plates. Wollina et al. (22) also showed differences in nail and matrix volume, also with higher levels in the male.

The results of our study showed that there is no significant difference between NU in the dominant and non-dominant hand. However, we would rather suspect that the dominant hand, as being more exposed to external factors, would have showed a higher thickness of NUs as an adaptation to more frequent exposure to destructive environmental factors and help in manipulation. Ruan et al. (23), however, also did not obtain data, suggesting differences between mentioned NU.

In our study, we found inverse statistically significant correlation between the age and the thickness of the nail plate only in the d5 in the whole study group and in the group of men (*r* = −0.3, *p* = 0.027; *r* = −0.4, *p* = 0.043, respectively), whereas there were no such observations in case of other fingers. Those correlations are most likely random statistical results. In general, in examined subjects with no evident onychopathy, there were no significant changes in nail thickness, which is commonly described in the elderly (24, 25).

### TOWL

Although the TEWL measurement is a well-recognized parameter reflecting the function of the stratum corneum (SC), this measurement in relation to the entirely keratinized structure of the nail plate, known as TOWL, is still not fully understood. There are just single reports regarding TOWL measurement within healthy NU, which present different methodology (6, 16, 26).

We have made an attempt to analyze the value of TOWL, taking into account various variables. We did not observe the relations of TOWL to age and sex, and the value of TOWL did not differ between the right and the left hand. We found that the lowest measured TOWL value was recorded in the group of men in d1 (1.10 c.u.), and the highest value was also observed in the group of men in nd3 (17.60 c.u.). Statistically significant correlations were found between finger type and TOWL value in the whole group and taking into account gender (*p* < 0.05) except of the non-dominant hand in men. In general, the further from the thumb the NU is, the higher TOWL values are obtained. We suspect that these findings may be linked to a better adaptation to preserve water in thumb and index, because of their utility in everyday activities. The results obtained in male non-dominant hand should be considered as random statistical discovery.

We observed moderate statistically significant correlation between the age and TOWL value only in the male group in the d3 finger (*r* = −0.5, *p* = 0.031) and we did not gain any statistically significant differences in the TOWL values of the corresponding types of fingers between male and female NUs. Jemec et al. (6) also showed no differences in case of TOWL—gender relation, but suggested that with aging TOWL, value decreases. However, they published data based on examination of thumbs' TOWL of 21 healthy volunteers (12 female, nine male; 22–71 years, median 32). We presume that differences between sexes may be related to the thicker nail plates in men; however, this observation was not confirmed by our research. Also, age may act as a factor deteriorating the function of water resistance within the NUs, although obtained data (TOWL value of only one type of NUs in men inversely correlated with age) suggest that it could be a random statistical finding.

We did not observe any significant statistical differences between NUs of the dominant and non-dominant hand. However, our suspicion was that the dominant hand (being highly exposed to external factors) would show a difference, with higher TOWL values. Murdan et al. measured TOWL values in only three volunteers using an evaporimeter with a closed condensation chamber with a dedicated adapter and showed that there are differences in the TOWL values in the same person in the corresponding fingers of the left and right hand and the left and right foot. However, the authors did not define if the extremities were dominant or non-dominant (16).

Also, the study of Sattler et al. (27) faces limitations considering the number of analyzed NUs—only a middle finger of the dominant and non-dominant hand was examined. The number of participants was higher than in previously mentioned studies; however, the group of 30 volunteers would benefit from broadening. In their study the authors used an open-chamber evaporimeter with holding ring. The researchers did not observe statistically significant differences in the TOWL values of the middle finger of the dominant and non-dominant hand, as it was presented in our study.

An equipment is definitely an important factor influencing acquired results. According to The European Group on Efficacy Measurement and Evaluation of Cosmetics and other Products (EEMCO) guidance for TEWL (17), three different measurement methods may be applied: open-chamber methods, semiopen methods (open-chamber method with wind shield incorporated in probe), and closed-chamber methods. Open-chamber evaporimeters are still the preferred techniques to measure TEWL. However, because of their susceptibility to environmental factors, they have been supplemented with semiopen- and closed-chamber probes, which are more convenient to use and more applicable to field studies. Closed-chamber methods interfere with evaporation and cannot be used for continuous monitoring. The authors suggest that validation of methods with respect to intra- and inter-instrument variation is still a challenge (17). Still, no guidelines related to TOWL measurement exist.

### Correlation Between HF-USG Results and TOWL Values

The results of previous studies based on assessment of correlation between nail plate thickness and water loss (measured using various methods) are inconclusive and based on small groups (6, 15, 16).

We found that thickness of the nail plate is not correlated with TOWL value in most cases. There was no statistically significant correlation between the TOWL value and the thickness of the nail plate measured with HF-USG in any of the tested NUs. A similar observation was also made by Jemec et al. (6); however, they assessed only thumbs. Moreover, Spruit (15) investigated the specific permeability of the nail plate to water. The thickness of all NUs was measured with a caliper in only one volunteer. The author concluded that in a healthy volunteer, the loss of water is independent of the thickness of the nail plate. On the other hand, different results, partially obtained *in vitro*, were presented by Murdan et al. They showed a relationship between TOWL value and plate thickness assessed by measuring cut nails with a micrometer (16).

According to previous research, TEWL is not affected by skin thickness (28). It is worth considering if, similarly, the water permeability of nail plates, either does not depend on the thickness of the SC. However, the differences in chemical composition may be the factor influencing permeability of SC in the skin (29). Is it true for the NU? More research is required. Certainly, it would be necessary to continue research in this area with a larger number of NU.

## Conclusions

To sum up, non-invasive methods, such as high-frequency ultrasonography and TEWL, enable assessment of the NU and are useful in examination of its structure and function. High-frequency ultrasonography shows characteristic elements of NU that can be distinguished because of differences in their echogenicity. The thickness of the nail plate and TEWL depend on the type of finger, and show a relationship with gender.

## Data Availability Statement

The raw data supporting the conclusions of this article will be made available by the authors, without undue reservation.

## Ethics Statement

The studies involving human participants were reviewed and approved by Bioethical Commission. The patients/participants provided their written informed consent to participate in this study.

## Author Contributions

RŻ and ZA assistance in writing article and performing research. AL statistical analysis. DJ supervision over writing thesis and English style analysis. AD-P assistance in conducting and interpreting research and supervision over writing thesis. AP originator of the research, assistance in conducting and interpreting research, author of the text on a par with the MS-L and AP. MS-L performer of research and analysis and author of the text on a par with the AP. All authors contributed to the article and approved the submitted version.

## Conflict of Interest

The authors declare that the research was conducted in the absence of any commercial or financial relationships that could be construed as a potential conflict of interest.
